# Identification of Genetic Determinants and Enzymes Involved with the Amidation of Glutamic Acid Residues in the Peptidoglycan of *Staphylococcus aureus*

**DOI:** 10.1371/journal.ppat.1002508

**Published:** 2012-01-26

**Authors:** Teresa A. Figueiredo, Rita G. Sobral, Ana Madalena Ludovice, João Manuel Feio de Almeida, Nhat K. Bui, Waldemar Vollmer, Hermínia de Lencastre, Alexander Tomasz

**Affiliations:** 1 Laboratory of Molecular Genetics, Instituto de Tecnologia Química e Biológica da Universidade Nova de Lisboa, Oeiras, Portugal; 2 Centro de Recursos Microbiológicos (CREM), Departamento de Ciências da Vida, Faculdade de Ciências e Tecnologia, Universidade Nova de Lisboa, Quinta da Torre, Caparica, Portugal; 3 Departamento de Ciências da Vida, Faculdade de Ciências e Tecnologia, Universidade Nova de Lisboa, Quinta da Torre, Caparica, Portugal; 4 Centre for Bacterial Cell Biology, Institute for Cell and Molecular Biosciences, Newcastle University, Newcastle upon Tyne, United Kingdom; 5 The Rockefeller University, Laboratory of Microbiology, New York, New York, United States of America; Dartmouth Medical School, United States of America

## Abstract

The glutamic acid residues of the peptidoglycan of *Staphylococcus aureus* and many other bacteria become amidated by an as yet unknown mechanism. In this communication we describe the identification, in the genome of *S. aureus* strain COL, of two co-transcribed genes, *murT* and *gatD*, which are responsible for peptidoglycan amidation. MurT and GatD have sequence similarity to substrate-binding domains in Mur ligases (MurT) and to the catalytic domain in CobB/CobQ-like glutamine amidotransferases (GatD). The amidation of glutamate residues in the stem peptide of *S. aureus* peptidoglycan takes place in a later step than the cytoplasmic phase – presumably the lipid phase - of the biosynthesis of the *S. aureus* cell wall precursor. Inhibition of amidation caused reduced growth rate, reduced resistance to beta-lactam antibiotics and increased sensitivity to lysozyme which inhibited culture growth and caused degradation of the peptidoglycan.

## Author Summary

Genetic determinants and enzymes that catalyze the multiple steps in the assembly of bacterial cell wall peptidoglycan have been known for some time. On the other hand, the mechanism by which glutamic acid residues of this structure undergo modification to glutamine has remained unknown. In this communication, we describe the identification of two genetic determinants that appear to be responsible for the completion of the chemical structure of the cell wall of the important human pathogen *S. aureus*. The availability of a conditional mutant which allows modulation of this system has allowed us to recognize the importance of glutamine residues for optimal growth rate and drug resistance and sensitivity of the staphylococcal peptidoglycan to the host defense factor lysozyme.

## Introduction

Peptidoglycan forms an essential stress-bearing and shape-maintaining layer in the bacterial cell envelope. Its biosynthetic pathway is the target of important classes of antimicrobials such as beta-lactams and glycopeptides, and the polymerized cell wall is targeted by antimicrobial enzymes like lysozyme. The biosynthesis of peptidoglycan is a complex process involving several consecutive enzymatic steps that take place in the cytoplasm and on the inner and outer surface of the cytoplasmic membrane. The cytoplasmic stage of biosynthesis culminates in the formation of the UDP-*N*-acetylmuramic acid (UDP-MurNAc) covalently linked to a pentapeptide which is composed of L-alanine, D-iso-glutamic acid, L-lysine (or *meso*-diaminopimelic acid, DAP) and D-alanyl D-alanine. The assembly of this stem peptide moiety involves a superfamily of enzymes, the Mur ligases [1]. In the next steps of biosynthesis, the UDP-MurNAc-pentapeptide is attached to a membrane acceptor undecaprenyl phosphate (C55-P) followed by the addition of GlcNAc to the MurNAc residues yielding the structure known as lipid II. Lipid II, i.e., the bactoprenol linked disaccharide pentapeptide is then transported to the outer surface of the cytoplasmic membrane where it serves as a substrate for polymerization reactions catalyzed by transpeptidases and transglycosylases to form the polymeric cell wall peptidoglycan.

Chemical analysis of the *S. aureus* peptidoglycan showed that the structure of these polymers differed from the structure of the cytoplasmic disaccharide pentapeptide cell wall precursor: some hydroxyl groups in the glycan chain were acetylated; and the second amino acid residue of the muropeptides was not iso-glutamic acid but its amidated version, iso-glutamine.

The mechanisms of these secondary modifications of the cell wall are not well understood. Enzymes and genetic determinants involved with the acetylation of the glycan chain and the role of this structural modification in the resistance of *S. aureus* against host lysozyme - have only been described recently [2].

While amidation of the stem peptide residues at positions 2 or 3 or both is frequent among gram-positive bacteria, the physiological roles of this chemical modification have remained a matter of speculation [3] and the genetic determinants and enzymes responsible for the conversion of iso-glutamic acid to iso-glutamine residues have also remained unknown.

In this communication we describe the identification of a small operon composed of two genes – *murT* and *gatD* – in the genome of the beta-lactam resistant *S. aureus* strain COL. Amino acid sequence of the protein products of these genes show similarity to murein ligases (*murT*) and to CobB/CobQlike glutamine amidotransferases (*gatD*). The properties of a conditional mutant of *murT/gatD* indicate that this operon is responsible for the conversion of isoglutamic acid to iso-glutamine residues in the peptidoglycan of *S. aureus*.

## Materials and Methods

### Bacterial strains, plasmids, and growth conditions

Bacterial strains and plasmids used in this study are listed in Table 1. *Staphylococcus aureus* strains were grown at 37°C with aeration in tryptic soy broth (TSB) or tryptic soy agar (TSA) (Difco Laboratories, Detroit, Mich.). Transposition mutant RUSA208 [4] and the conditional mutant strains RN4220*pCadmurT-gatD* and COL*pCadmurT-gatD*, the double mutant RUSA208*pCadmurT-gatD*, the complemented strains COL*pCadmurT-gatD*+pSK*murT* and COL*pCadmurT-gatD*+pSK*gatD* and the control strain COL*pCadmurT-gatD*+pSK were grown in the presence of the respective antibiotics (Table 1). The growth medium was supplemented with 0.2 µM of cadmium chloride (CdCl_2_; Sigma, St. Louis, MO), unless otherwise described.

**Table 1:** Strains and plasmids used in this study.

Strain or plasmid	Description	Source or reference
**Strains**		
*S. aureus*		
RN4220	Mc^s^; restriction negative	(R. Novick)
COL	Homogeneous Mc^r^ (MIC, 1600 ìg/ml); Em^s^	Rockefeller University Collection
RUSA208	COL with Tn*551* insertion in *glnR*, Em^r^	[4]
COL*pCadmurT-gatD*	COL with *murT-gatD operon* under *Pcad* control, Kan^r^, Neo^r^	This study
COL*pCadmurT*-*gatD*+pSK*murT*	COL*pCadmurT-gatD* with pSK5632 plasmid with *murT* gene, Kan^r^, Neo^r^ Cm^r^	This study
COL*pCadmurT*-*gatD*+pSK*gatD*	COL*pCadmurT-gatD* with pSK5632 plasmid with *gatD* gene, Kan^r^, Neo^r^ Cm^r^	This study
COL*pCadmurT-gatD*+pSK	COL*pCadmurT-gatD* with pSK5632 plasmid, Kan^r^, Neo^r^, Cm	This study
RUSA208*pCadmurT-gatD*	COL with an insertion of Tn*551* in *glnR*, Ery^r^ and with *murT-gatD* operon under *Pcad* control, Kan^r^, Neo^r^	This study
*E. coli*		
DH5á	*recA endA1 gyrA96 thi-1 hsdR17 supE*44 *relA1* F80 D*lac*ZDM15	Invitrogen
**Plasmids**		
pBCB20	*S. aureus* integrative vector with *Pcad* inducible promotor, Ap^r^, Kan^r^	R.Sobral and M.Pinho (unpublished)
pMurT′	pBCB20 a vector with *murT* rbs and the first 298 codons fused to *Pcad* promotor, Ap^r^, Kan^r^	This study
pSK5632	*E. coli*-*S. aureus* shuttle vector, Ap^r^, Cm^r^	[13]
pSK*murT*	pSK5632 vector with *murT* gene and 300 bps of the immediately upstream region, Ap^r^, Cm^r^	This study
pSK*gatD*	pSK5632 vector with *gatD* gene, Ap^r^, Cm^r^	This study

*Escherichia coli* strains (Table 1) were grown in Luria-Bertani broth (LB; Difco Laboratories) with aeration at 37°C. Erythromycin (10 µg/ml), neomycin sulphate (50 µg/ml), kanamycin (50 µg/ml), chloramphenicol (10 µg/ml) and ampicillin (100 µg/ml) from Sigma were used for the selection and maintenance of *S. aureus* and *E. coli* mutants.

### In silico analysis of the *murT-gatD* gene products

The amino acid sequences of ORFs SACOL1951 (MurT) and SACOL1950 (GatD) were retrieved from the UniProtKB database [5], and their domain architecture was checked using the InterProScan tool [6]. The domains were aligned through TCoffee [7]. Given the limited similarity between sequences, secondary structure inference was used as an independent benchmark for the alignment. This inference was accomplished through Psipred [8]. Position specific annotation other than the one present in the InterPro documentation was collected from references [1], [9].

### DNA methods

Restriction enzymes from New England Biolabs (Beverly, MA) were used as recommended by the manufacturer. Routine PCR amplification was performed with *Tth* DNA polymerase (HT Biotechnology, Cambridge, United Kingdom) and PCR amplification for cloning purposes was performed using Pfu DNA polymerase (Stratagene, Heidelberg, Germany).

For plasmid DNA extraction High pure Plasmid Purification Kit (Roche, Basel, Switzerland) was used. PCR and digestion products were purified using High pure PCR Purification Kit (Roche). Ligation reactions were performed using Rapid DNA Ligation kit (Roche).

### Reverse transcription analysis

Reverse transcription (RT)-PCR was performed as described [10] using total RNA from strain COL as the template. The primers used for the reverse transcription reactions are described in Table 2 and the amplification conditions were: 94°C for 2 min; 40 cycles of 94°C for 30 s, 53°C for 30 s, and 72°C for 2 min; and one final extension step of 72°C for 5 min.

**Table 2:** Reduced monomeric muropeptides in HPLC fractions analyzed by LTQ-FT mass spectrometry.

Peak N°	Proposed muropeptide structure(s)^1^	Theoretical neutral mass (Da)	Determined neutral mass (Da)
**I** a	**Tetra(Gln)Gly_3_**	1067.4983	1067.5227
	**Tetra(Gln)Gly_4_**	1124.5197	1124.5410
	Tetra(Gln)Gly_5_	1181.5412	1181.5290
	Tetra(Gln)Gly_6_	1238.5626	1238.5933
	Tetra(Gln)Gly_7_	1295.5841	1295.6296
**II** b	**Penta(Gln)Gly_2_**	1081.5139	1081.5242
	Penta(Gln)Gly_3_	1138.5354	1138.5427
	Penta(Gln)Gly_4_	1195.5568	1195.5806
**III** b	**Penta(Gln)Gly_3_**	1138.5354	1138.5423
	Penta(Gln)Gly_4_	1195.5568	1195.5801
**IV** b	**Penta(GlcNAc)(Gln)Gly_2_**	1284.5933	1284.6128
	Penta(GlcNAc)(Gln)Gly_3_	1341.6147	1341.6536
	Penta(GlcNAc)(Gln)Gly_4_	1398.6362	1398.6787
**V** c	**Tetra(Glu)Gly_3_**	1068.4823	1068.4794
	Tetra(Glu)Gly_4_	1125.5037	1125.5191
	Tetra(Glu)Gly_5_	1182.5252	1182.4912
**VI** c	**Penta(Glu)Gly_2_**	1082.4979	1082.5122
	Penta(Glu)Gly_3_	1139.5194	1139.5170

1 Muropeptides with main MS intensities are in bold.

a Structures found in strain COL and in COL*pCadmurT* grown with 0 and 0.2 µM CdCl_2_.

b Structures found in strain COL and in COL*pCadmurT* grown with 0.2 µM CdCl_2_.

c Structures found in COL*pCadmurT* with 0 µM CdCl_2_.

### Construction of pMurT′ plasmid

A 918- bp DNA fragment of *murT* gene was amplified by PCR using chromosomal DNA from strain COL as a template and the specific primers PmurT′-R and PmurT′-F (Table 2). The amplification conditions used were as follows: 94°C for 4 min; 30 cycles, each consisting of 94°C for 30 s, 50°C for 30 s, and 72°C for 1 min 30 s; and one final extension step of 72°C for 10 min. The amplified fragment and the integrative plasmid pBCB20, carrying a cadmium chloride inducible promoter (R.G. Sobral and M.G. Pinho, unpublished) were both digested with SmaI and ligated, generating plasmid pMurT′.

### Construction of the conditional mutant

Plasmid pMurT′ was electroporated into competent cells of RN4220 with a Gene Pulser apparatus (Bio-Rad, California) under conditions described previously [11]. Selection of the transformants was performed using kanamycin (50 µg/ml), neomycin sulphate (50 µg/ml) and 0.2 µM of Cadmium chloride. The correct insertion of pMurT′ into RN4220 chromosome was confirmed by PCR, using an internal *murT* primer chosen outside the region cloned and an internal pBCB20 primer (Table 2). The *murT-gatD* conditional mutation was then transduced, by phage 80α to the background of COL as previously described [12] and mutant COL*pCadmurT-gatD* was obtained.

### Construction of complemented strains

A 1673 bp DNA fragment, including the complete *murT* coding sequence and 300 bp of the immediate upstream region was amplified from COL genome using the primers P*murT*SalI and P*murT*BamHI (Table 2). The amplified *murT* fragment and plasmid pSK5632 [13] were digested with SalI and BamHI and ligated, generating the replicative plasmid pSK*murT*. The same strategy was used for the construction of the replicative plasmid pSK*gatD*, in which a 1088 bp DNA fragment including the complete *gatD* gene sequence and 300 bp of the immediately upstream region.

Plasmids pSK*murT* and pSK*gatD* were separately introduced into RN4220 by electroporation and subsequently transferred to COL*pCadmurT-gatD* by transduction, generating COL*pCadmurT-gatD*+pSK*murT* and COL*pCadmurT-gatD*+pSK*gatD*, respectively. Plasmid pSK5632 was also introduced in the conditional mutant, providing the control strain COL*pCadmurT-gatD*+pSK.

### Construction of RUSA208*pCadmurT-gatD* double mutant

The *murT-gatD* conditional mutation was transduced, using phage 80α, to the background of RUSA208. The obtained double mutant RUSA208*pCadmurT-gatD*, has a transposon insertion in *glnRA* operon and the *murT-gatD* operon under the control of *pCad* promoter.

### Northern blotting analysis

Cells were grown in TSB at 37°C to mid-exponential phase (OD_620 nm_ of 0.7). Prior to harvesting the cells, the RNA protect reagent (QIAGEN, Hilden, Germany) was added to the cultures. Total RNA was isolated as previously described [14]. PCR amplified internal fragments of the *murT, gatD*, SACOL1949-SACOL1948, SACOL1952, *glnA* and *pta* genes were used as probes for hybridization (the primers used are listed in Table S1). The DNA probes were labeled with [α-^32^P]dCTP (Perkin Elmer, MA, USA).

### Cell wall isolation

Isolation of cell wall was performed as described [2], [15]. Briefly, cells were harvested by centrifugation, washed twice with cold 0.9% NaCl, resuspended in 0.9% NaCl and boiled for 20 min. After chilling on ice, the suspension was centrifuged and washed twice with 0.9% NaCl. The cells were disrupted using 106 µm glass beads (Sigma) and FastPrep FP120 apparatus (Bio 101, La Jolla, Calif.), purified, washed, and boiled for 30 min in 5% SDS, diluted in 50 mM Tris/HCl pH 7, to remove non-covalently bound proteins. After centrifugation, the cell wall fragments were diluted in 0.1 M Tris-HCl (pH 6.8) and incubated with 0.5 mg/ml trypsin for 16 h at 37°C to degrade cell-bound proteins. Purified cell walls were washed with double-distilled water and lyophilized.

### Peptidoglycan purification

Lyophylised cell wall was treated with 49% of hydrofluoric acid for 48 hours at 4°C in order to remove teichoic acids. The teichoic acid free peptidoglycan was washed with water several times to remove all traces of hydrofluoric acid and then lyophylised.

### Peptidoglycan analysis by RP-HPLC

Identical amounts of peptidoglycan were digested with mutanolysin (1 mg/ml; Sigma). The resulting muropeptides were reduced with sodium borohydride and separated by reverse-phase-high performance liquid chromatography (RP-HPLC) using a Hypersil ODS (Runcorn Cheshire, UK) column (3 µm particle size, 250×4.6 mm, 120 Å pore size) and a linear gradient from 5% to 30% MeOH in 100 mM sodium phosphate buffer pH 2.5 at a flow rate of 0.5 ml/min as described [16].

### Purification of monomeric muropeptides

Highly purified cell wall was prepared as previously described [2] and resuspended to a final concentration of 10 mg/ml. Cell wall material (500 µg) was digested with lysostaphin (300 µg) in 20 mM amonium acetate, pH 4.8, for 24 h at 37°C with stirring. Subsequently, cellosyl (Höchst AG, Frankfurt, Germany) (15 µg) was added to the reaction mixture which was incubated for 12 h at 37°C. Finally, additional 15 µg of cellosyl was added and the incubation continued for an additional 12 h. The enzymatic reaction was stopped by boiling the samples for 5 min and insoluble contaminants were removed by centrifugation. The digested cell wall was reduced with sodium borohydride and the resulting monomeric muropeptides were separated by RP-HPLC using a Prontosil (Bischoff, Leonberg, Germany) column (3 µm, particle size, 250×4.6 mm, 120 Å pore size), and a linear gradient from 0% to 30% MeOH in 10 mM sodium phosphate buffer pH 6.0 at a flow rate of 0.5 ml/min.

### Mass spectrometry analysis of monomeric muropeptides

The eluted fractions corresponding to the most predominant peaks of the chromatograms were collected after HPLC separation, concentrated to 10–20 µl, and acidified with 1% trifluoroacetic acid (TFA). The samples were then desalted and further concentrated using ZipTips (C- 18, Millipore, UK) according to the standard protocol recommended by the manufacturer. The material was eluted from the ZipTip with 3 µl of 50% acetronitrile, 0.1% TFA and was sprayed directly into a Finnigan LTQ-FT mass spectrometer (Thermo, Bremen, Germany) operating in positive mode (Pinnacle Proteomics Facility, Newcastle University, UK) [17].

### Analysis of UDP-linked precursor pool

The UDP-linked peptidoglycan precursors from the cytoplasmic pool were isolated using a modified protocol [18]. Briefly, vancomycin (Sigma) was added (at five times the minimal inhibitory concentration) to mid-exponential grown cultures and incubation proceeded for additional 30 minutes. The cultures were then chilled below 10°C, cells were harvested, suspended in cold water and slowly stirred into the same volume of boiling water for 15 minutes. After centrifugation the supernatant was collected, lyophilized, dissolved in water and the pH was adjusted to 4.0 using 20% phosphoric acid. The suspension was again centrifuged and the pH of the supernatant adjusted to 2.0. The suspension was centrifuged at 4°C for 1 h at 200000 *g*.

The UDP-linked peptidoglycan precursors were separated through the same column used to separate the muropeptides of peptidoglycan – using a linear gradient from 0 to 30% of MeOH in 100 mM sodium phosphate buffer (pH 2.0), with a flow rate of 0.5 ml/min. Compounds to be analyzed by MS were isolated and desalted using the same column as before with a linear gradient from 0 to 30% of MeOH in 10 mM of sodium phosphate (pH 4.3) for 25 min with a flow rate of 0.5 ml/min. Mass spectral data were obtained by MALDI-TOF analysis (Pinnacle Proteomics Facility, Newcastle University, UK).

### Growth curves

Overnight grown cultures of strains COL and COL*pCadmurT-gatD*, COL*pCadmurT-gatD*+pSK*murT*, COL*pCadmurT-gatD*+pSK*gatD* and COL*pCadmurT-gatD*+pSK were diluted 1∶1,000 into fresh TSB supplemented with the respective antibiotics (Table 1). The conditional mutants were grown in media containing CdCl_2_ concentrations at 0, 0.01, 0.05 and 0.2 µM. The cultures were incubated at 37°C with agitation and the OD_620_ nm was monitored over time.

### Determination of beta-lactam resistance

Overnight grown cultures of strains COL and COL*pCadmurT-gatD*, COL*pCadmurT-gatD*+pSK*murT* and COL*pCadmurT-gatD*+pSK*gatD* and COL*pCadmurT-gatD*+pSK were plated on TSA supplemented with increasing concentrations of CdCl_2_ (0, 0.01, 0.05 and 0.2 µM) and incubated at 37°C for 24 hours. Oxacillin (Sigma) diffusion disks (1 mg) were used to determine inhibition halos.

### Turbidometric assay of peptidoglycan hydrolysis

To analyze the susceptibility of peptidoglycan to lysozyme hydrolysis, a turbidometric assay was used as described [2], [19]. Briefly, 0.5 mg of purified peptidoglycan from the conditional mutant, grown with and without CdCl_2_, were sonicated in 1 ml of 100 mM Sodium-Potassium phosphate buffer pH 6.6. Human lysozyme or hen egg white lysozyme (Sigma) was added to a final concentration of 300 µg/ml and the reaction was incubated at 37°C. The optical density was monitored at 660 nm.

### Determination of lysozyme and polymyxin resistance

The impact of lysozyme on exponential growth was determined as described [19]. Overnight cultures of the conditional mutant grown with inducer were diluted to an OD_620 nm_ of 0.1 in fresh TSB (with and without inducer). The cultures were incubated at 37°C until an OD_620 nm_ of 1.0. Then, each culture was diluted 1∶10 into fresh TSB medium and lysozyme (300 µg/ml) was added at an OD_620 nm_ of 1.0. The growth was monitored for several hours.

The same procedure was done using 20 µg/ml of Polymyxin B (Sigma), a cationic antimicrobial peptide.

## Results

The two open reading frames, SACOL1951 and SACOL1950, were automatically annotated in the genome of *S. aureus* strain COL as a putative Mur ligase family-like protein and a CobB/CobQ-like glutamine amidotransferase, respectively. The preliminary annotations of these genes, designated *murT* and *gatD*, respectively, suggested a role for their protein products in cell wall metabolism.

### *murT* and *gatD* genes are co-transcribed as a small operon

**Figure 1:**
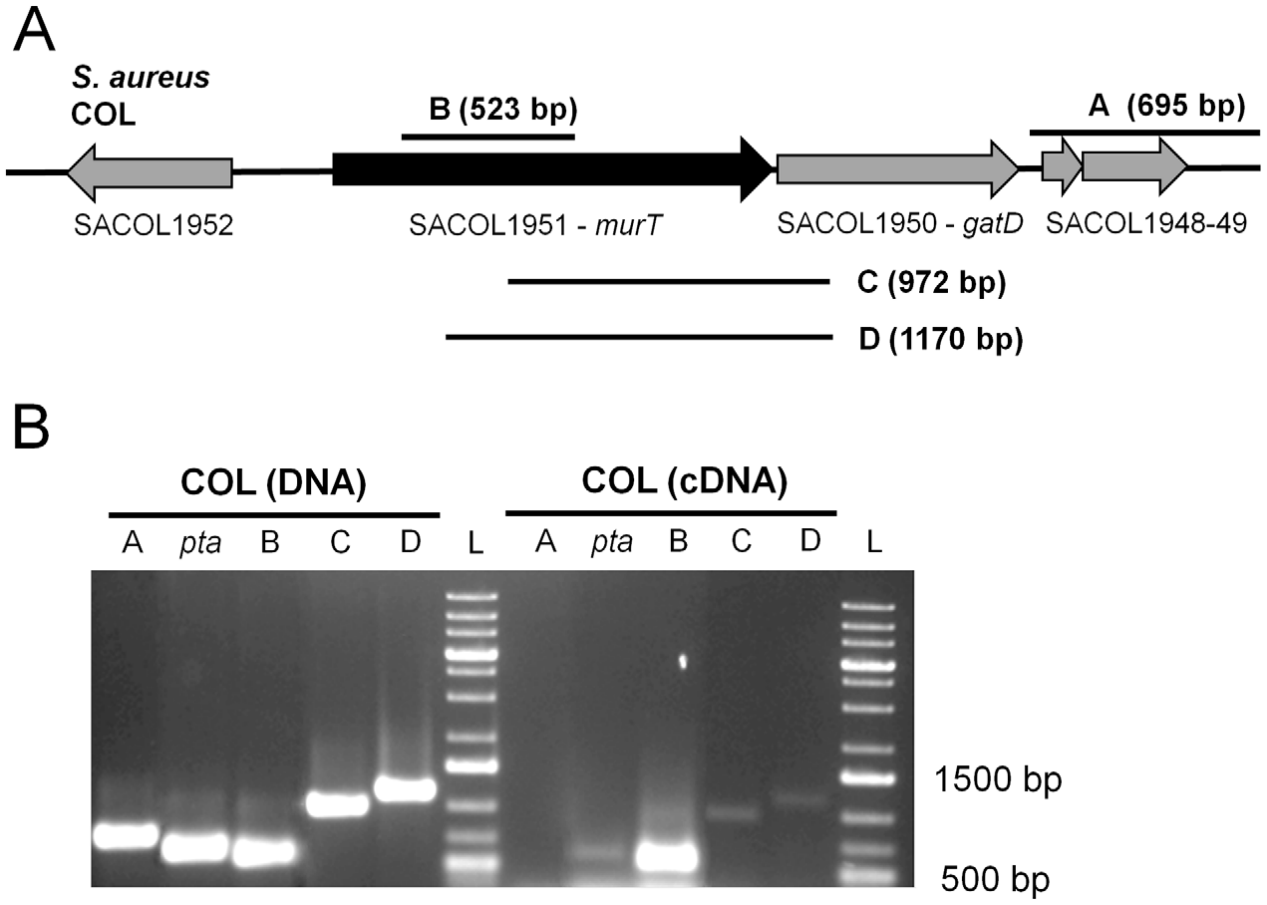
RT-PCR amplification of the *murT-gatD* region. (**A**) *S. aureus* COL genome region encompassing *murT* and *gatD* genes and the vicinity regions. Fragments A, B, C, and D which were amplified by RT-PCR using primers from Table S1, are shown. (**B**) Amplification results by PCR using COL DNA and by RT-PCR using COL cDNA produced from a total RNA sample. A fragment from *pta* gene was used as a positive internal control. Fragment A was used as a negative control, as no transcript was detectable for SACOL1948 and SACOL1949 by northern blotting (data not shown).

DNA sequence analyses of *murT-gatD* region suggested that *murT* and *gatD* are located in the same operon and might be co-transcribed from a common promoter: the *murT* stop codon and the *gatD* methionine codon are separated by 4 bp only; both genes are transcribed in the same direction and no promoter region sequence could be found upstream from *gatD* (Figure 1A).

Reverse transcription-PCR (RT-PCR) was performed using total cDNA of strain COL with forward primers specifically binding to *murT* and a reverse primer specifically binding to *gatD*. The test yielded products of the expected size (Figure 1B, lanes C and D). No PCR product was obtained from the negative control using primers from the SACOL1949-1948 region, which was found by northern blotting not to be transcribed (Figure 1B, lane A). A PCR product of the expected size was obtained for the positive control, using primers internal to *pta*, a housekeeping gene.

The results of the RT-PCR test indicated that both *murT* and *gatD* are co-transcribed from a common promoter.

### The *murT-gatD* operon is a syntenic block

Analysis of genome sequences available showed that the *murT* and *gatD* genes occur, widespread among bacteria, as a syntenic block, although it is not a universal feature. This is in agreement with our RT-PCR results, which identified the two genes as a small operon. The distribution of this syntenic block among the prokaryotes, with emphasis on the *Staphylococcaceae*, is shown in Figure S1.

### Construction of a *murT-gatD* conditional mutant

In order to explore the functions of these uncharacterized genes we constructed a mutant strain containing a single chromosomal copy of *murT-gatD* under the control of an inducible promoter (*pCad*). A DNA fragment of *murT* gene which includes the first 298 codons and the ribosome binding site but not the promotor region, was cloned into the integrative plasmid pBCB20 (see Table 1). The recombinant plasmid was electroporated into RN4220 and the chromosomal construct was transduced into the background of the MRSA strain COL. The only complete functional copies of *murT* and *gatD* genes were located immediately downstream from the *pCad*, generating mutant COL*pCadmurT-gatD* (Figure S2). Hence, this strain expresses the *murT-gatD* genes when grown in the presence of Cd^2+^, and both genes are depleted when Cd^2+^ is absent from the growth medium (see below).

### Transcriptional analysis of the *murT-gatD* conditional mutant

Northern blotting assays were performed in order to confirm the specificity of transcription of the *murT-gatD* operon controlled by the CdCl_2_ concentration in the medium. The transcription of *murT*, *gatD*, SACOL1952 and SACOL1948-SACOL1949 genes was analyzed for COL and mutant COL*pCadmurT-gatD* grown with several concentrations of inducer. The level of *murT* and *gatD* transcription was found to increase with the inducer concentration in the medium (data not shown). No alterations were detected under the same conditions in the transcription level of the ORFs located in the immediate vicinity of the *murT-gatD* operon, SACOL1952 and SACOL1949-SACOL1948, which were found to be not transcribed even for strain COL (data not shown). The housekeeping gene *pta* was used as control.

For strain COL, a single transcript was visualized for each gene: an mRNA structure of approximately 1780 nt long hybridized with *murT* probe and an mRNA structure of approximately 2300 nt long was obtained for hybridization with *gatD* probe. The size of this last transcript matches the size of both genes, consistent with their co-transcription.

### Abnormal peptidoglycan produced upon *murT-gatD* depletion

**Figure 2:**
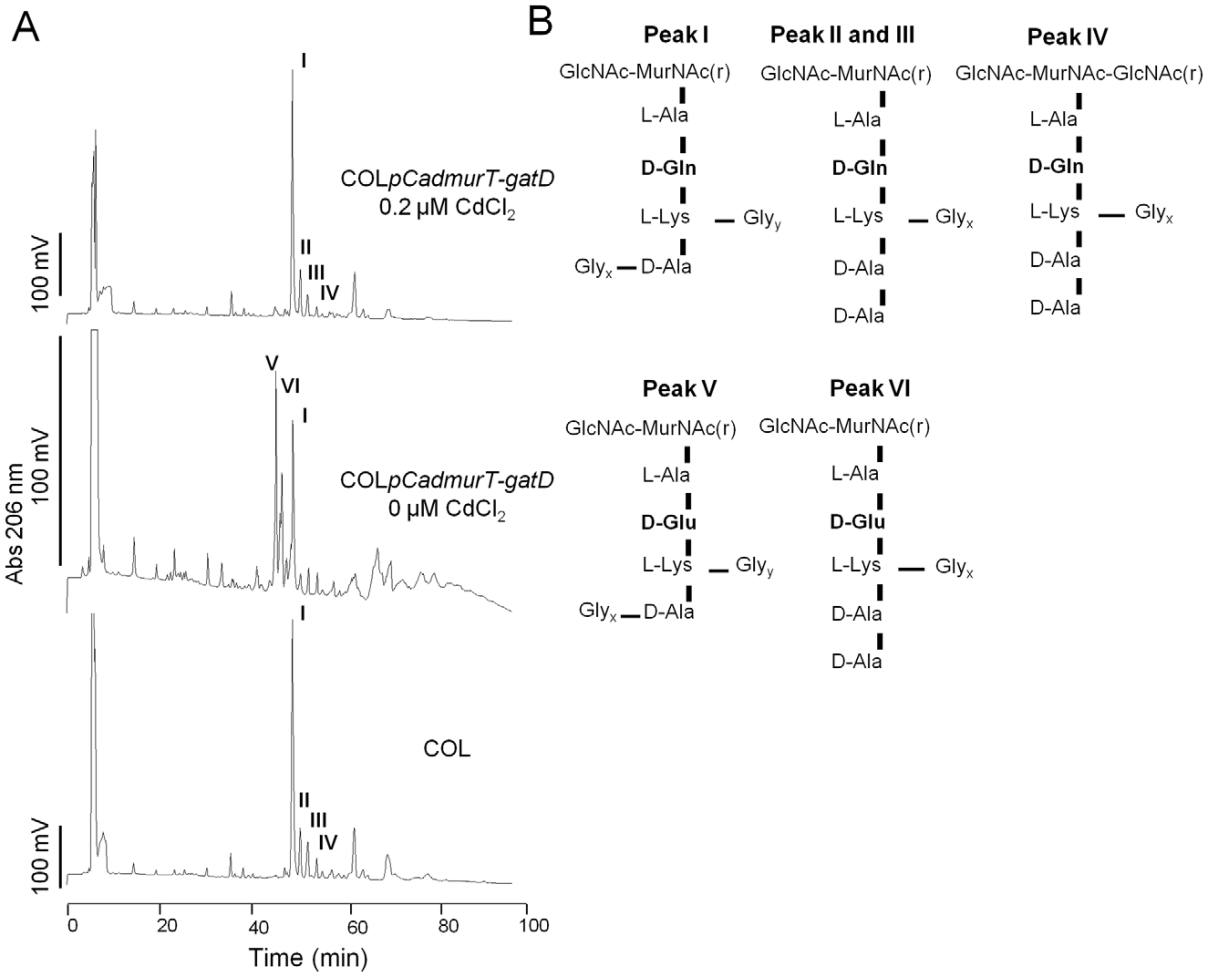
RP-HPLC profiles of cell walls of strains COL and the conditional mutant. (**A**) RP-HPLC profiles of cell walls prepared from strains COL and COL*pCadmurT-gatD* mutant grown with and without 0.2 µM of CdCl_2_. The cell walls were purified and digested with cellosyl and lysostaphin and the resulting muropeptides were reduced and analyzed by RP-HPLC. Fractions eluting at 50.73 min (peak I), 52.42 min (peak II), 54.10 min (peak III), 56.13 min (peak IV) in COL strain, and eluting at 47.00 min (peak V) and 48.33 min (peak VI) in COL*pCadmurT-gatD*, grown without the inducer, were collected and analyzed by mass spectrometry. (**B**) Proposed structures for the muropeptides corresponding to peaks I, II, III, IV, V and VI. Structures with different numbers of glycine residues associated with the D-Ala and L-Lys of the stem peptide, were identified for each peak. The mass of the analyzed compounds are presented in Table 2.

Cell walls of parental strain COL and of the conditional mutant, grown with and without Cd^2+^, were purified and digested with cellosyl and lysostaphin. The resulting monomeric muropeptides were reduced and analyzed by RP-HPLC. The muropeptide profiles revealed that, when the transcription of *murT-gatD* operon was inhibited, two new muropeptide structures appeared in the RP-HPLC profile (Figure 2A – peaks V and VI). These two muropeptide species showed shorter retention times than peak I, which is common to all the profiles. To identify the structural modifications, all peaks annotated in Figure 2A were isolated and analyzed by MS.

The MS results (Table 2) indicate that the two new peaks (V and VI) observed in the profile of the *murT/gatD* depleted cells corresponded to muropeptide structures with D-iso-glutamate in the stem peptide replacing D-iso-glutamine. Peaks I, II, III and IV correspond to muropeptide structures with D-iso-glutamine (Figure 2B).

Amidated muropeptides (Peak I) were still present when the transcription of *murT-gatD* operon was inhibited. This could be due to the activity of MurT and GatD expressed by residual transcription from the *pCad* promoter or to the presence of other enzymes with the same activity. These findings identify the protein products of *murT-gatD* as essential for the full amidation of the D-glutamic acid residues in the *S. aureus* peptidoglycan.

### Comparison of the peptidoglycan composition of the *murT-gatD* mutant and *glnRA* mutant

**Figure 3:**
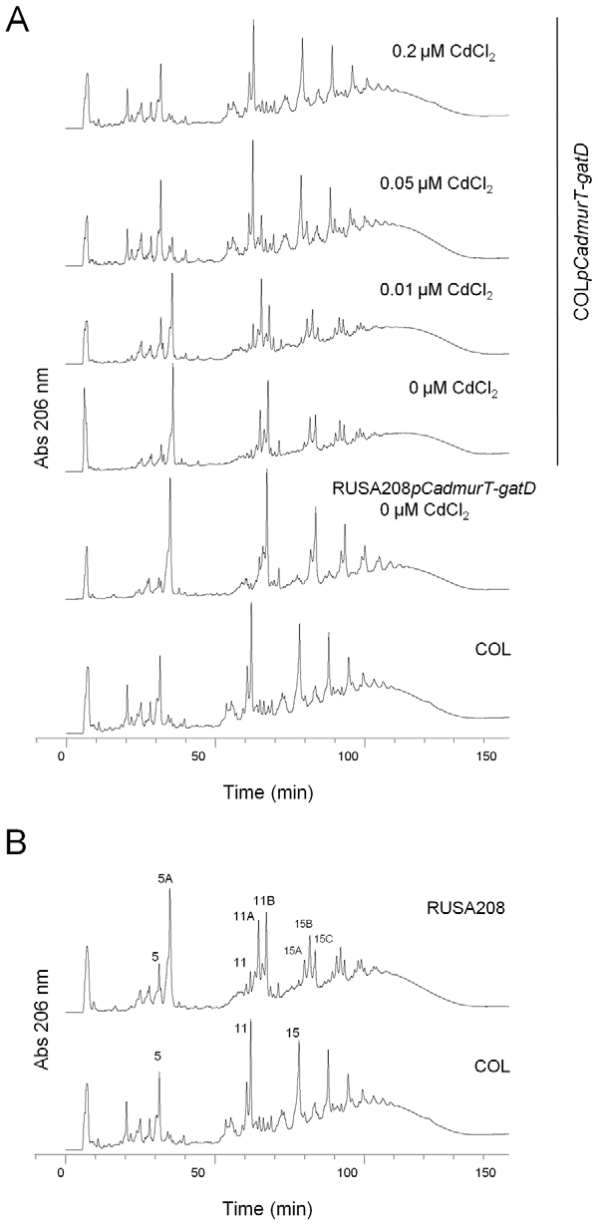
RP-HPLC cell wall profiles. The purified peptidoglycan was digested with mutanolysin, reduced and analyzed by RP-HPLC. (**A**) muropeptide profiles of strains COL, RUSA208*pCadmurT-gatD*, grown without CdCl_2_, and COL*pCadmurT-gatD* grown with 0, 0.01, 0.05 and 0.2 µM of CdCl_2_. (**B**) muropetide profiles of strains COL, RUSA208. The muropeptide structures corresponding to peaks 5, 5A, 11, 11A, 11B, 15, 15A, 15B and 15C were inferred from mass spectrometric analysis (4).

The cell walls of the parental strain COL and the conditional mutant COL*pCadmurT-gatD* grown with different concentrations of inducer were extracted, the peptidoglycan purified, digested with muramidase and the muropeptides analyzed by RP-HPLC (Figure 3A).

The elution profile of the conditional mutant grown in the absence of CdCl_2_ showed longer retention times for all peaks, when compared with COL. In addition, the peaks corresponding to muropeptide structures with higher oligomerization level (retention time over 60 min) were split into two or more smaller peaks eluting at very similar retention times. The elution profiles of the mutant grown with 0.01 µM and 0.05 µM of CdCl_2_ showed gradual re-establishment of the parental muropeptide pattern. For cells grown in 0.2 µM CdCl_2_ supplemented medium, the optimal inducer concentration, the peptidoglycan HPLC profile was indistinguishable from that of strain COL.

The muropeptide elution profile of COL*pCadmurT-gatD*, grown in the absence of inducer, showed similarities to the elution profile of the previously characterized *glnRA* transposition mutant RUSA208 [4] (Figure 3B). In RUSA208, the transposon inserted into the *glnR* gene which codes for the repressor of the glutamine synthetase operon *glnRA*, resulting in the abolishment of *glnA* transcription.

The impact of the *glnRA* mutation on the peptidoglycan of RUSA208 has been described as the substitution of the normal D-iso-glutamine residues by D-iso-glutamic acid at position 2 of the stem peptide [4]. Substitution of iso-glutamine by iso-glutamic acid residues has been observed among muropeptide monomers (Peak 5A in Figure 3B), among dimeric muropeptides (Peaks 11A & 11B in Figure 3B), among the tripeptide structures (Peak 15B in Figure 3B) and among three of the stem peptides represented by peaks 15A, B & C in Figure 3B. All these structures are also present in the conditional mutant COL*pCadmurT-gatD* grown in the absence or at suboptimal concentrations of the inducer (see Figure 3A).

The *glnA* gene sequence in COL*pCadmurT-gatD* was identical to that in strain COL, excluding the possibility that a mutation in *glnA* causes the deficiency in peptidoglycan amidation as it occurs in RUSA208 strain. Also, the transcription of the *glnA* gene did not vary with the Cd^2+^ concentration in COL*pCadmurT-gatD* (data not shown) discarding the hypothesis that *murT* and/or *gatD* may indirectly reduce *glnA* transcription.

The peptidoglycan profiles of RUSA208 and COL*pCadmurT-gatD* grown with no CdCl_2_, showed that amidation of the muropeptides still occured partially. This may be due to a leaky expression of *murT-gatD* operon through *pCad* promoter in the absence of CdCl_2_. In the case of RUSA208, other sources of amino group, besides glutamine, may be used, although less efficiently.

The peptidoglycan HPLC profile of the double mutant RUSA208*pCadmurT-gatD* showed a virtually complete lack of amidated muropeptides (Figure 3A), indicating that the gene products of these two operons are together needed for the amidation of the glutamic acid residue of the peptidoglycan.

### Complementation of the *murT-gatD* conditional mutation

The transcriptional analysis showed that the expression of both *murT* and *gatD* genes is being controlled in COL*pCadmurT-gatD* mutant, through the concentration of inducer added to the medium. For this reason we constructed two independent complementation mutants, COL*pCadmurT-gatD*+pSK*murT* and COL*pCadmurT-gatD*+pSK*gatD*, by separately introducing into the COL*pCadmurT-gatD* mutant, the replicative plasmid pSK5632 with either the *murT* or the *gatD* gene. Cloning of the *murT*-*gatD* operon into pSK5632 was also attempted, but this construct did not yield viable *E. coli* transformants. Strain COL*pCadmurT-gatD*+pSK harboring pSK5632 with no cloned gene was constructed and used as control.

### Re-establishment of the normal peptidoglycan composition in the *murT-gatD* complementation mutants

With the two complementation strains available, we obtained three distinct levels of re-establishment of the normal peptidoglycan: i) the in trans complementation with several copies of the *murT* gene showed a partially restored peptidoglycan with a small amount of muropeptides containing glutamic acid residues (COL*pCadmurT-gatD*+pSK*murT* – 0 µM CdCl_2_, Figure S3A); ii) the in trans complementation with several copies of *gatD* gene showed no re-establishment of the normal peptidoglycan profile (COL*pCadmurT-gatD*+pSK*gatD* – 0 µM CdCl_2_, data not shown); iii) the in trans complementation with several copies of the *murT* gene and sub-optimal expression of the chromosomal copy of *murT-gatD* operon showed complete restoration of the peptidoglycan profile (COL*pCadmurT-gatD*+pSK*murT* – 0.01 µM CdCl_2_, Figure S3B). In the latter case (iii), the 0.01 µM CdCl_2_ of added inducer is responsible for providing a sub-optimal number of copies of *murT-gatD* transcripts, adding to the already available copies of *murT* transcript provided in trans. The few copies of *gatD* provided in this condition are enough for a complete re-establishment of the normal peptidoglycan composition. Thus, complementation of the *murT*-*gatD*-depletion phenotype requires the expression of *murT* and at least a basal level of *gatD*.

### Composition of cell wall precursor pool of COL*pCadmurT-gatD* mutant

In order to identify the biosynthetic stage at which amidation occurred, the cell wall precursor pool was analyzed by RP-HPLC from strains COL and for the *murT-gatD* conditional mutant grown with and without the inducer. The HPLC profiles were identical for the three conditions analyzed (Figure S4). The major peak, eluting at 38 minutes, was isolated from the cytoplasmic fractions of COL and of the *murT-gatD* conditional mutant grown with and without the inducer. The corresponding structures were analyzed by MALDI-TOF MS. The results indicated an identical molecular mass of 1149.35 (neutral mass) for each of the three samples, consistent with the structure of the UDP-MurNAc-L-Ala-D-iGlu-L-Lys-D-Ala-D-Ala, the last cytoplasmic precursor. The presence of D-iso-glutamate in these three structures indicated that the conversion of glutamic acid to iso-glutamine residues must occur at a later stage of cell wall precursor biosynthesis – most likely in the lipid phase – confirming an earlier finding [20].

### Properties of the conditional mutant

#### Deficit in growth rate

**Figure 4:**
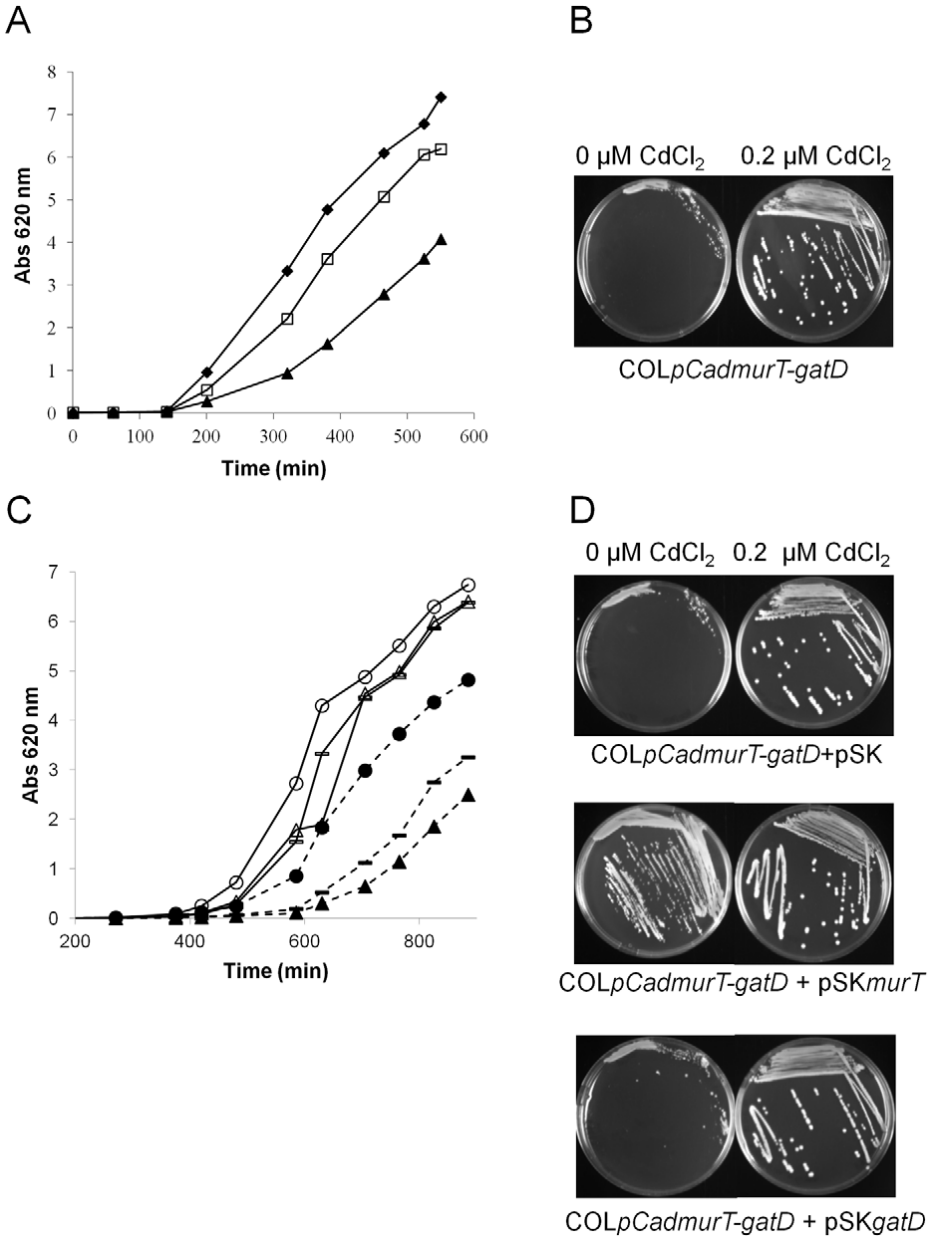
Growth rate of the conditional mutant at different concentrations of CdCl_2_. (**A**) Growth curves of strains COL (black diamond), and the COL*pCadmurT-gatD* conditional mutant in TSB supplemented with (black triangle) 0 µM of CdCl_2_ and (white square) 0.2 µM of CdCl_2_. (**B**) Growth on solid medium with or without supplementation of 0.2 µM CdCl_2_ of COL*pCadmurT-gatD*. (**C**) Growth curves in liquid medium supplemented with Cm (10 µg/ml) of the complementation mutants COL*pCadmurT-gatD*+pSK*murT* with 0.2 µM CdCl_2_ (white circle) or without CdCl_2_ (black circle), COL*pCadmurTgatD*+pSK*gatD* with 0.2 µM CdCl_2_ (white triangle) or without CdCl_2_ (black triangle), and the control strain COL*pCadmurT-gatD*+pSK with 0.2 µM CdCl_2_ (white line) or without CdCl_2_ (black line). (**D**) Growth on solid medium, with or without supplementation of 0.2 µM CdCl_2_, of the complementation strains COL*pCadmurT-gatD*+pSK*murT*, COL*pCadmurT-gatD*+pSK*gatD*, and the control strain with the plasmid pSK5632, COL*pCadmurT-gatD*+pSK.

The *murT-gatD* depleted cells had normal morphology as examined by electron microscopy (data not shown) but their growth rate was greatly reduced, indicating that the amidation of peptidoglycan is required for normal growth. COL*pCadmurT-gatD* was unable to grow on solid medium in the absence of Cd^2+^. In liquid medium the growth rate was significantly reduced in the absence of Cd^2+^, and it increased with the concentration of inducer added to the medium (Figure 4A and 4B).

The growth rate of the COL*pCadmurT-gatD*+pSK*murT* strain in the absence of inducer was higher than the growth rate of the control strain COL*pCadmurT-gatD*+pSK, although a complete restoration could not be obtained. In contrast, the growth rate of the COL*pCadmurT-gatD*+pSK*gatD* strain was lower than that of the control strain COL*pCadmurT-gatD*+pSK (Figure 4C). This behavior was more obvious when the strains were grown in solid medium (Figure 4D).

#### Decrease in beta-lactam resistance

**Figure 5:**
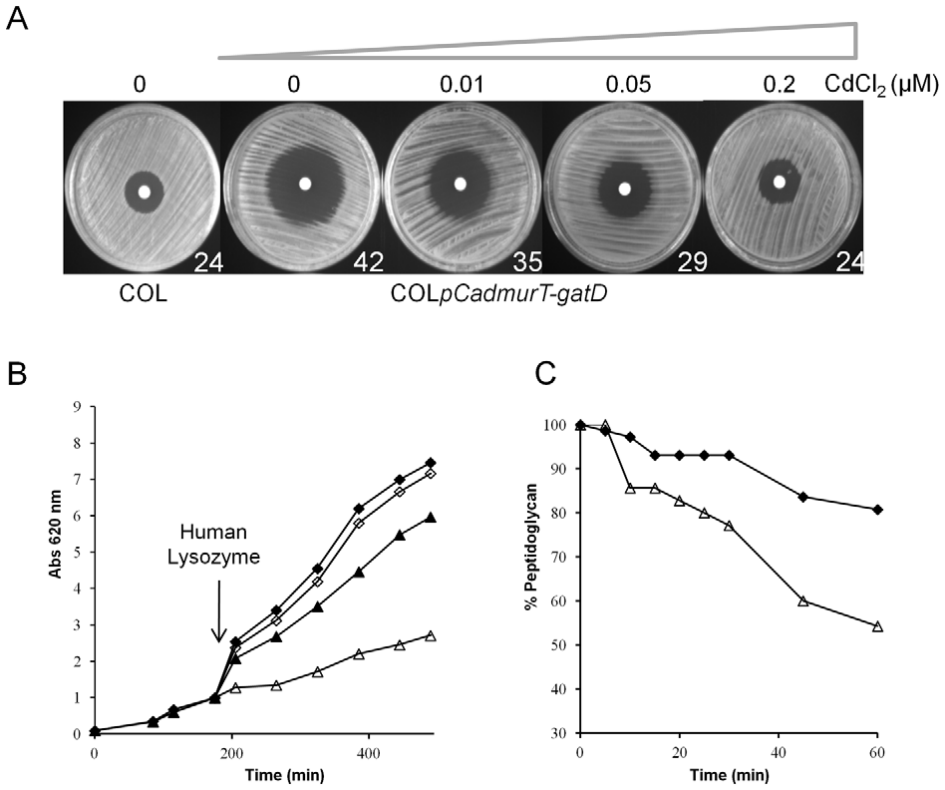
Reduced antibiotic resistance and increased sensitivity to lysozyme in the conditional mutant. (**A**) Oxacillin inhibition halos (1-mg oxacillin disks) were determined for COL and COL*pCadmurT-gatD* mutant grown with 0, 0.01, 0.05 and 0.2 µM of CdCl_2_. At the right hand side of each dish is the diameter of the respective inhibition halo in mm. (**B**) Effect of human lysozyme on the growth rate of: COL*pCadmurT-gatD* grown with 0.2 µM of CdCl_2_ (black diamond), COL*pCadmurT-gatD* grown with 0.2 µM of CdCl_2_+lysozyme (white diamond), COL*pCadmurT-gatD* grown with 0 µM of CdCl_2_ (black triangle), COL*pCadmurT-gatD* grown with 0 µM of CdCl_2_+lysozyme (white triangle). Human lysozyme was added (300 µg/ml) at an OD_620 nm_ of 1.0 for all strains (arrow). (**C**) Effect of human lysozyme (300 µg/ml) on peptidoglycan purified from COL*pCadmurT-gatD* grown with 0.2 µM of CdCl_2_ (black diamond), or COL*pCadmurT-gatD* grown with 0 µM of CdCl_2_ (white triangle).

The oxacillin resistance level of COL*pCadmurT-gatD* was found to depend on the inducer concentration: as the CdCl_2_ concentration was reduced, the size of the growth inhibition halos increased (Figure 5A). In the presence of 0.2 µM of CdCl_2_, the resistance phenotype was identical to that of COL. COL*pCadmurT-gatD*+pSK*murT*, grown in the absence of inducer, completely re-established the parental phenotype (data not shown).

#### Increased sensitivity to lysozyme

The *murT-gatD* depleted cells of COL*pCadmurT-gatD* grown in the absence of Cd^2+^ were sensitive to human lysozyme (Figure 5B), while the same cells grown in the presence of Cd^2+^ were lysozyme resistant, as was the parental strain COL (data not shown), indicating that peptidoglycan amidation is required to express lysozyme resistance. By contrast, *murT-gatD* depleted cells did not show diminished resistance to the cationic antimicrobial peptide polymyxin B (data not shown). Next, the sensitivity to lysozyme of peptidoglycan of the conditional mutant grown in the absence and presence of inducer were compared. After an incubation period of 60 minutes, lysozyme was able to hydrolyse 46% of peptidoglycan from the conditional mutant grown in the absence of inducer. For the mutant grown with inducer, less than 20% of peptidoglycan was hydrolysed (Figure 5C).

*MurT-gatD*-depleted cells and peptidoglycan isolated from them showed also increased susceptibility to hen egg white lysozyme (data not shown).

### Amino acid sequence analysis of MurT and GatD

MurT shares approximately 15% identity and 53% similarity with the sequence of the Mur ligases of *S. aureus*. Interestingly, while MurT shares the characteristic Mur ligase central domain [1], [21] as defined at InterPro (IPR013221), Pfam (PF08245) and Panther (PTHR23135) MurT lacks the flanking N- and C-terminal domains (Figure S5A).

Among the conserved residues were some critical motifs required for ATP and Mg^2+^ binding and other conserved sites that may not be directly involved in catalysis (Figure S5A). In addition, the MurT protein has a C-terminal domain of unknown function (Pfam: DUF1727, InterPro: IPR013564), which is also found at the C-terminus of more than 900 sequences of prokaryotic proteins at UniProt, and in 5 different domain architectures, all of them sharing the same ORF, or in contiguous ORFs, with Mur central domain (PF08353).

GatD shows similarity to one of the two domains of a cobyric acid synthetase protein: a glutamine-dependent amidotransferase (Gn-AT), with glutamine amide transfer (GAT) activity. Its architecture comprises the overlapping domain signatures of CobB/CobQ_GATase (InterPro: IPR017929), and GATase_3 (InterPro: IPR011698) domains. Through multiple sequence alignment of the N-terminal region of three known Gn-ATs, the absence of a large fragment was noted in GatD (Figure S5B). This missing fragment included important residues for the dethiobiotin synthase activity [9] and part of the ATP binding motif. By placing the representation of the secondary structures over the sequence alignment, we can observe considerable agreement between the shared regions, especially near the reactive center of GATase_3 (Figure S5B). This domain harbored the conserved residues directly involved in GAT activity, according to IPR011698. GatD was also found to contain the unusual Triad family glutamine amidotransferase domain with conserved Cys and His residues (Figure S5B), but lacking the Glu residue of the catalytic triad, as the CobB and CobQ proteins [9].

## Discussion

The basic structure of *S. aureus* peptidoglycan is known to undergo at least two major secondary modifications, the *O*-acetylation of the free OH groups in the glycan strand and the amidation of the γ-carboxyl group of the second residue of the stem peptide, D-iso-glutamate, resulting in the formation of D-iso-glutamine. *O*-acetylation of the *S. aureus* peptidoglycan confers lysozyme resistance to the bacteria and its main genetic determinant, the *oatA* gene has been identified and characterized recently [2].

In contrast, the mechanism of the amidation of glutamic acid residues has remained unknown.

In this communication we report the identification of two genetic determinants – *murT* and *gatD* – in the genome of *S. aureus* strain COL - that are required and sufficient for peptidoglycan amidation. A conditional mutant constructed for these two genes, showed abnormal peptidoglycan composition, with decreased amidation of the glutamate residue. The characterization of a double mutant in which not only the expression of *murT-gatD* operon is inhibited but also the operon *glnRA*, responsible for providing glutamine substrate, is impaired, allowed us to infer that *murT* and *gatD* are the key determinants for the amidation of *S. aureus* peptidoglycan.

Furthermore, through the analysis of the precursor pool composition of the mutant strain we showed that this modification step does not occur in the cytoplasm and most probably takes place at the membrane level, confirming previous observations [20].

Other phenotypes associated with *murT-gatD* mutation are decreased growth rate, decreased resistance to beta-lactams and to lysozyme hydrolysis.

The strong impact on growth rate suggests that an amidated peptidoglycan may provide better substrates for proteins that catalyze peptidoglycan biosynthesis and cell division. Lack of the amide group may create an unbalance between the synthetic and the hydrolytic machineries of the cell. Electron microscopy pictures of the conditional mutant showed cells with normal size. However, fewer cells showed complete septa, suggesting slower biosynthesis of the septum (data not shown).

The amidation of glutamic residues had already been shown to have a major impact on the expression of beta-lactam resistance, through the *femC (glnRA)* mutant of MRSA [22]. Consistent with this result, the depletion of *murT-gatD* also shows a major decrease in the oxacillin resistance level. The mechanism of this effect is not well understood [23].

However, similar effects were already described for several other genes [23] many of them related to peptidoglycan biosynthesis. One of the existing theories is that the structurally abnormal lipid II or cell wall peptides are poorer substrates for PBP2A.

More unexpectedly, another feature observed in this mutant was the decrease of resistance to lysozyme action. Lysozyme belongs to the innate immune response and acts on bacteria by hydrolyzing the β-1,4 glycosidic bonds between the two sugar molecules of the glycan strands of peptidoglycan (muramidase activity). Several cell wall modifications have been implicated in the lysozyme resistance mechanism of *S. aureus*, namely the *O*-acetylation in the C-6 position in the MurNac [2] and the presence of wall teichoic acids [24].

Firstly, we observed in vivo, that the mutant cells grown in the absence of inducer were susceptible to lysozyme action, as the growth was impaired. Besides muramidase activity, lysozyme has also cationic antimicrobial peptide (CAMP) activity [19]. The enhanced inhibitory action of lysozyme towards the mutant could be associated with either one of the two activities or both. However, we did not observe any effect of polymyxin B, a CAMP, on the growth rate of the mutant, indicating that glutamate amidation is important to prevent the muramidase activity of lysozyme. This effect could be a direct consequence of glutamate amidation or an indirect effect associated with changes in *O*-acetylation of the MurNac and/or in wall teichoic acids (WTA). For the mutant grown in the absence of inducer, the purified peptidoglycan, which lacks *O*-acetyl groups and WTA, suffered faster hydrolysis by lysozyme than normally amidated peptidoglycan. These observations allowed us to conclude that glutamate amidation is one of the key factors for lysozyme resistance in *S. aureus*.

The role of glutamate amidation has already been described in the context of pathogenesis. Peptidoglycan is sensed by the human innate immune system via NOD1 and NOD2 [25]; NOD1 recognizes as minimal structure the D-Glu-meso-DAP dipeptide, typical of Gram-negative bacteria, and is impaired by D-iso-glutamine presence suggesting the involvement of this modification in immune evasion. However, the same was not observed for NOD2, whose binding activity to muropeptides is not affected by the amidation of glutamic acid [26]. Also, this modification did not induce cytokine production, indicating that it is not involved in the modulation of pro-inflammatory capacity [27].

Amidation of peptidoglycan glutamic acid residue is common to many bacterial species – not all pathogenic (Figure S1) – suggesting additional physiological roles for this modification. One role of amidation could be to reduce the number of cell wall carboxylate groups that have recently been implicated together with wall teichoic acid phosphate residues to cooperatively bind divalent cations like Mg^2+^ or Mn^2+^ [28].

### A model for the cooperative functions of MurT and GatD

The *murT-gatD* operon emerged as a syntenic block that seems to be widespread among bacteria. Interestingly, for the distant taxa of *Actinobacteria*, in some rare cases, the two ORFs are merged into a single one (Figure S1).

**Figure 6:**
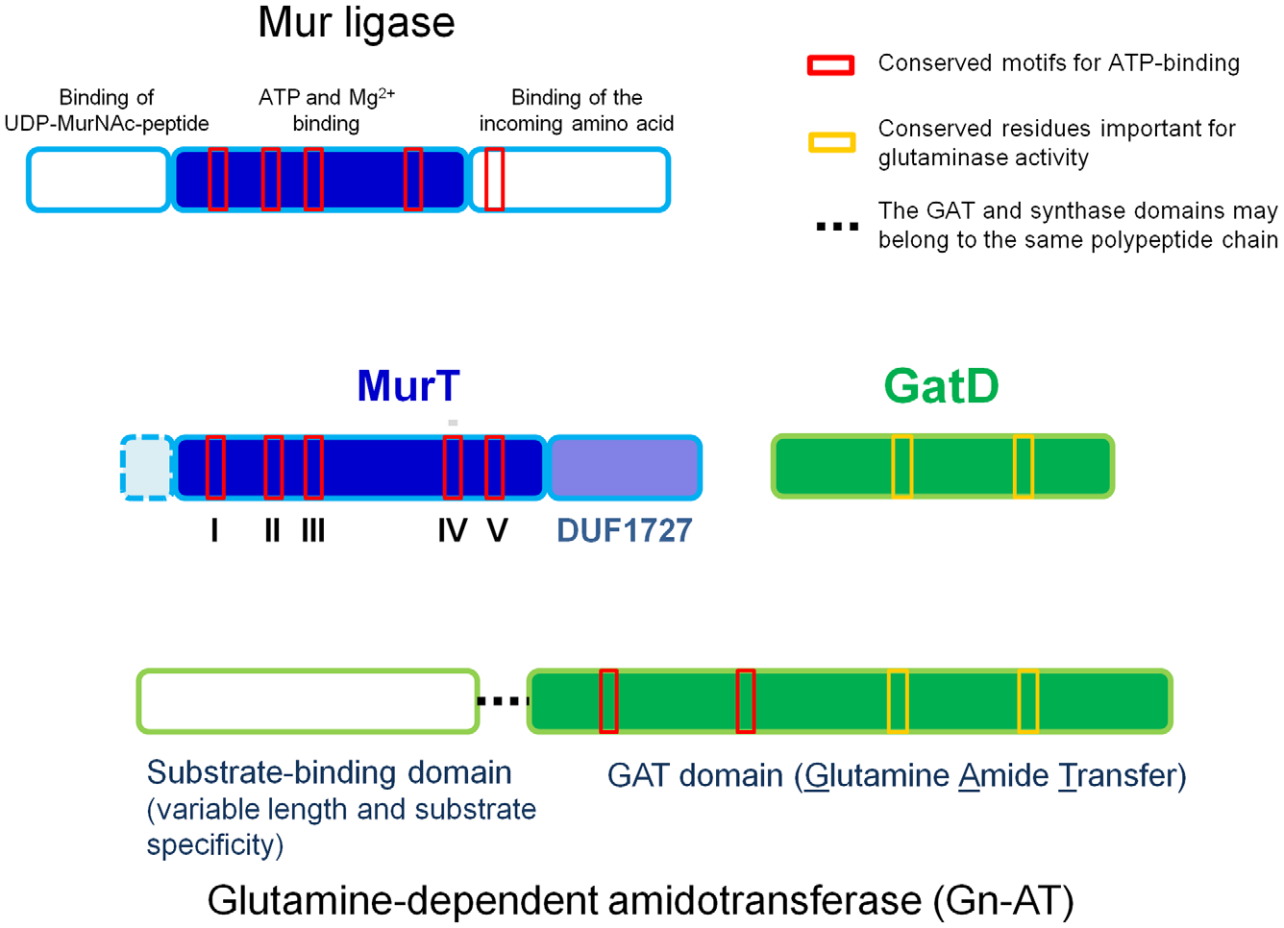
Protein regions necessary for the cooperative function of MurT and GatD proteins. The top panel represents the general topology of Mur ligase family proteins, with three domains. The only domain showing homology with MurT is the central domain involved in nucleotide binding. The conserved motifs for ATP-binding are indicated by red boxes. The lower panel represents the modular structure of Gn-ATs, with a synthase domain and a GAT domain which has glutaminase activity motifs (yellow boxes) and ATP-binding motifs (red boxes). GatD only shares the glutaminase motifs.

The genome co-localization of the two determinants, together with data from sequence analysis, led us to suggest a model for the coordinated function of MurT and GatD proteins in the peptidoglycan glutamate amidation (Figure 6).

Both proteins together harbor all domain functions required for amidation of peptidoglycan precursor: MurT may be responsible for the recognition of the reaction substrates, the lipid-linked peptidoglycan precursor and ATP, while GatD could be the catalytic subunit involved in the transfer of the amino group from free glutamine to the peptidoglycan precursor. The GatD sequence lacks an ATP binding motif which is common to all members of the Gn-AT family suggesting an activity that depends on the MurT protein which exhibits a typical Mur ligase central domain including the ATP binding motif (Figure 6).

Experiments are in progress to better define the roles of MurT and GatD proteins in the mechanism of amidation of *S. aureus* peptidoglycan precursor. Irrespective of mechanistic details, the results with the conditional mutant of *murT/gatD* clearly indicate that the amidation of glutamic acid residues in the *S. aureus* peptidoglycan is catalyzed by the concerted action of these two enzymes. The *murT-gatD* operon appears to be the last missing genetic determinant to account for the structural variation in the *S. aureus* peptidoglycan.

## Supporting Information

Figure S1 **Distribution of** ***murT/gatD*** **(red) among prokaryotes.** Bacillaceae is depicted in magenta because only one species has the pair. In grey are the taxonomic groups for which no sequence information is available. Actinobacteria present three cases of fused ORFs. The tree representation was built with the help of iTOL (http://itol.embl.de/), and it is based on the structure of the NCBI Taxonomy hierarchy. It should not be considered as a proper phylogenetic tree.
(PDF)

Figure S2 **Construction of the** ***murT-gatD*** **conditional mutant.** A 918 bp DNA fragment containing the ribosome binding site and the 5′sequence of SACOL1951 ORF was cloned downstream from *pCad* promoter. The resulting plasmid, pMurT′, was introduced into *S. aureus* RN4220 by electroporation and integrated into the chromosomal SACOL1951-1950 region by Campbell type recombination. The only complete copy of *murT-gatD* operon is under the control of the *pCad* promoter, in the COL*pCadmurT-gatD* conditional mutant.
(PDF)

Figure S3 **RP-HPLC profiles of purified peptidoglycan digested with mutanolysin.** (**A**) Comparison of peptidoglycan elution profiles of strains COL, mutant COL*pCadmurT-gatD* grown without inducer and the complementation strain COL*pCadmurT-gatD*+pSK*murT* grown without inducer. The complementation strain shows partial re-establishment of the abnormal amidation level. (**B**) Comparison of peptidoglycan elution profiles of strains COL, mutant COL*pCadmurT-gatD* grown without inducer, with sub-optimal inducer concentration (0.01 µM of CdCl_2_) and the complementation strain COL*pCadmurT-gatD*+pSK*murT* grown with 0.01 µM of CdCl_2_ and with 0.2 µM of CdCl_2_. The complementation strain grown with sub-optimal inducer concentration shows complete re-establishment of the abnormal amidation level.
(PDF)

Figure S4 **RP-HPLC profiles of UDP-linked precursor pools.** The UDP-linked precursor pools of the train COL and COL*pCadmurT-gatD* grown with or without 0.2 µM of CdCl_2_. The major precursor structure (elution time of 38.0 min) was identified by mass spectrometry as UDP-MurNAc-L-Ala-D-iGlu-L-Lys-D-Ala-D-Ala.
(PDF)

Figure S5 **Structure-informed aminoacid sequence alignments.** (**A**) Sequence alignmentof the central domain of Mur ligases. Residues involved with the nucleotide binding of four known *S. aureus* COL Mur ligases and MurT are labelled TP (ATP triphosphate), Mg1 and Mg2 (magnesium), A (adenine), and Ri (ATP ribose). The residue labelled C is the carbamoylated lysine residue observed in all the Mur enzymes except MurC; in this enzyme a glutamate residue, indicated with an asterisk (*) seems to play the same role in Mg2 coordination. The initial alignment was performed by TCoffee [7], the secondary structure was inferred for all sequences through Psipred [8], and the alignment was manually edited according to the latter.SACOL1951-MurT; SACOL1790-MurC; SACOL1196-MurD; SACOL1023-MurE; SACOL2073-MurF. In the top line α-helixes (green cylinders) and β-strands (orange arrows) were inferred for the sequences of the known Mur ligases. (**B**) Sequence alignment of the N-terminal halves of three known GATases. The residues involved in nucleotide binding of *S. aureus* COL GatD and three known GATases are indicated with a filled red box and are labelled TP (ATP triphosphate). Residues in green boxes marked with DS are deemed important for the dethiobiotin synthetase activity. The residues in the filled box labelled C are annotated as being directly involved with reactive center according to the GATase 3 (IPR011698) domain documentation. The initial alignment was performed by TCoffee [7], the secondary structure was inferred for all sequences through Psipred [8], and the alignment was manually edited according to the latter.GatD-Q5HEN2_STAAC (*S. aureus*); COBB_BACME (*Bacillus megaterium*); COBB_METJA (*Methanocaldococcus jannaschii*); COBB_PSEDE (*Pseudomonas denitrificans*). In the top line α-helixes (green cylinders) and β-strands (orange arrows) were inferred for the sequences of these three known GATases. In the bottom line, the same information is shown for GatD.
(PDF)

Table S1 **Primers used in this study.**
(DOC)
