# Supplementary figures and images for: Identification of Genetic Determinants and Enzymes Involved with the Amidation of Glutamic Acid Residues in the Peptidoglycan of Staphylococcus aureus
Source: PLoS Pathog. 2012 Jan 26;8(1):e1002508. doi: 10.1371/journal.ppat.1002508 (PMC3267633; doi:10.1371/journal.ppat.1002508)

Figure S1

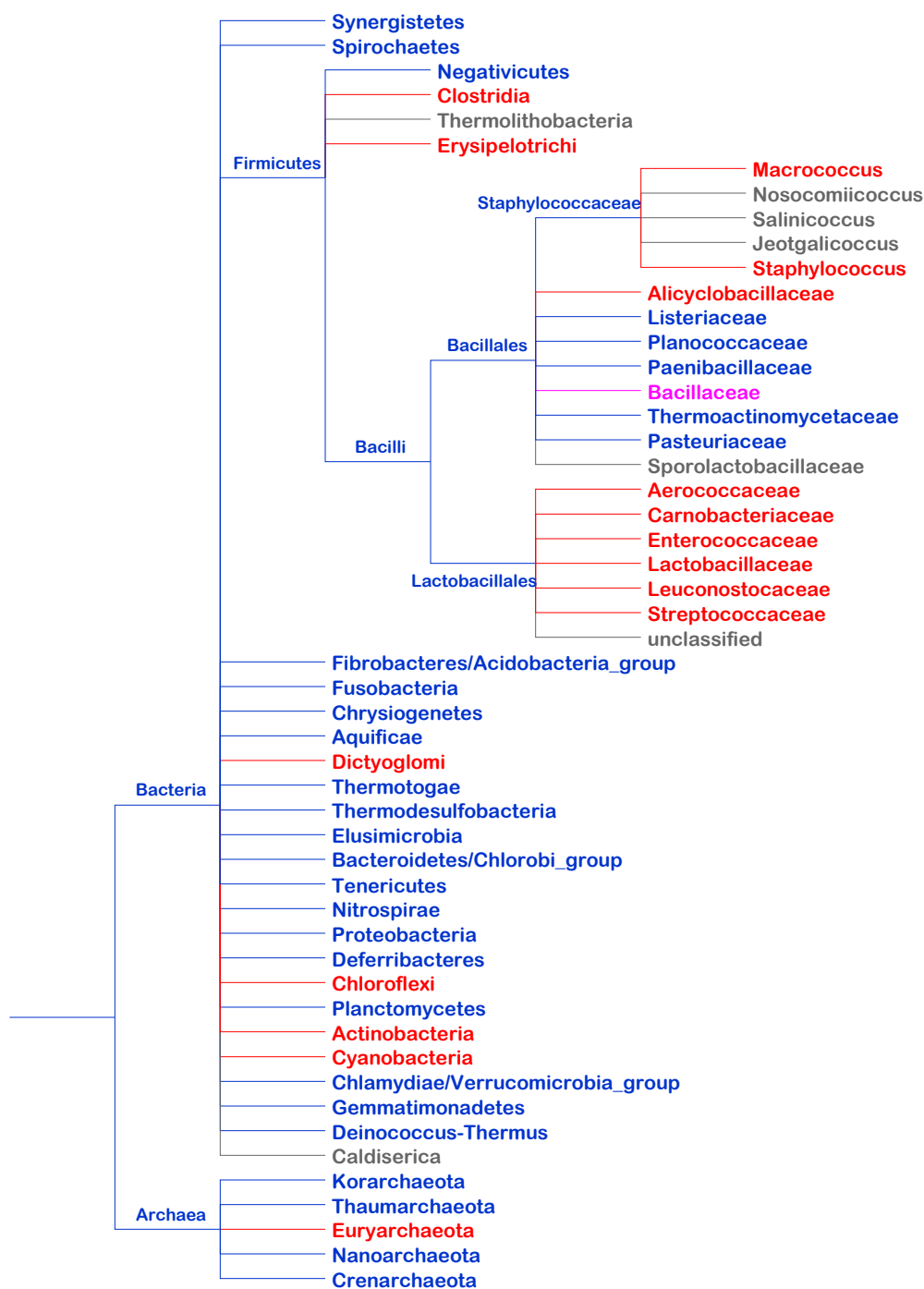

Supplement: Figure S1 — Distribution of murT/gatD (red) among prokaryotes. Bacillaceae is depicted in magenta because only one species has the pair. In grey are the taxonomic groups for which no sequence information is available. Actinobacteria present three cases of fused ORFs. The tree representation was built with the help of iTOL (http://itol.embl.de/), and it is based on the structure of the NCBI Taxonomy hierarchy. It should not be considered as a proper phylogenetic tree. (PDF) [file ppat.1002508.s001.pdf]

Figure S2

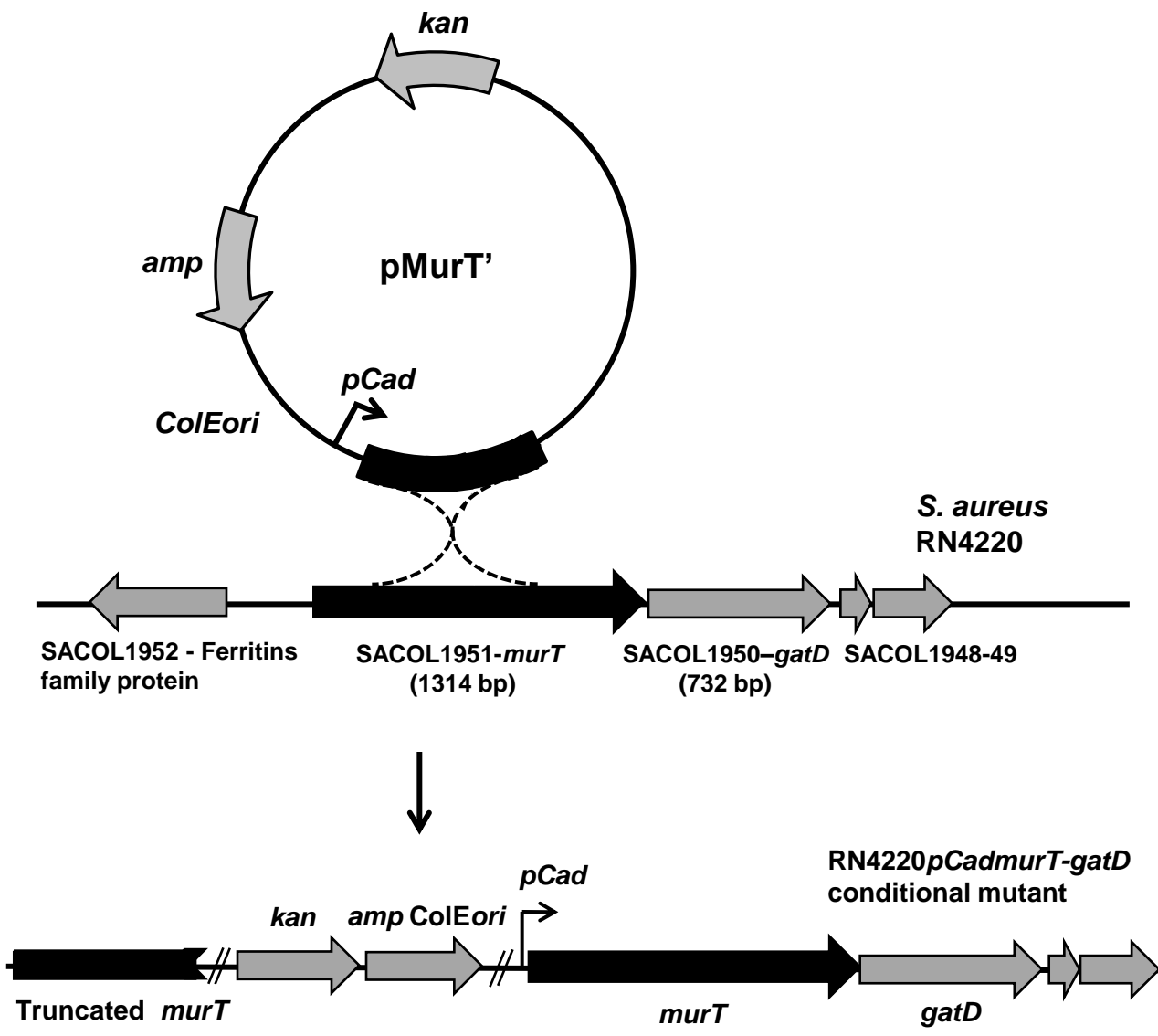

Supplement: Figure S2 — Construction of the murT-gatD conditional mutant. A 918 bp DNA fragment containing the ribosome binding site and the 5′sequence of SACOL1951 ORF was cloned downstream from pCad promoter. The resulting plasmid, pMurT′, was introduced into S. aureus RN4220 by electroporation and integrated into the chromosomal SACOL1951-1950 region by Campbell type recombination. The only complete copy of murT-gatD operon is under the control of the pCad promoter, in the COLpCadmurT-gatD conditional mutant. (PDF) [file ppat.1002508.s002.pdf]

Figure S3

**A**

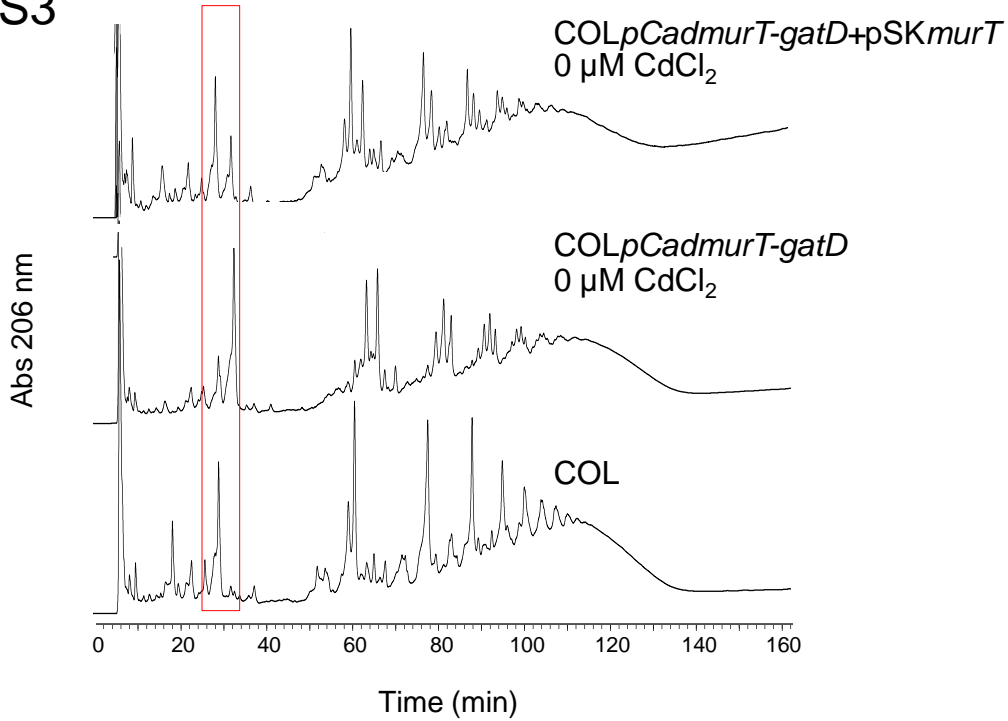

**B**

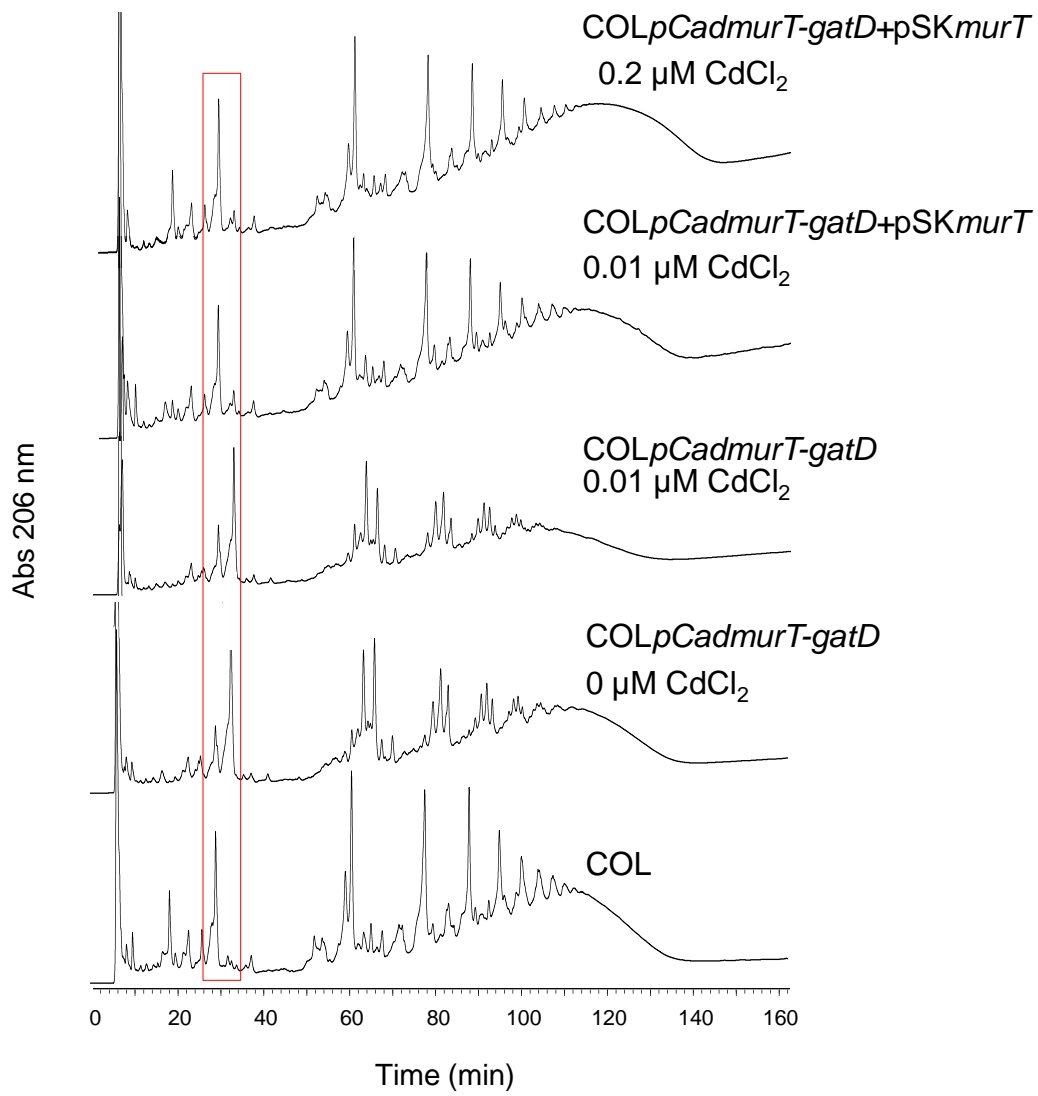

Supplement: Figure S3 — RP-HPLC profiles of purified peptidoglycan digested with mutanolysin. (A) Comparison of peptidoglycan elution profiles of strains COL, mutant COLpCadmurT-gatD grown without inducer and the complementation strain COLpCadmurT-gatD+pSKmurT grown without inducer. The complementation strain shows partial re-establishment of the abnormal amidation level. (B) Comparison of peptidoglycan elution profiles of strains COL, mutant COLpCadmurT-gatD grown without inducer, with sub-optimal inducer concentration (0.01 µM of CdCl2) and the complementation strain COLpCadmurT-gatD+pSKmurT grown with 0.01 µM of CdCl2 and with 0.2 µM of CdCl2. The complementation strain grown with sub-optimal inducer concentration shows complete re-establishment of the abnormal amidation level. (PDF) [file ppat.1002508.s003.pdf]

Figure S4

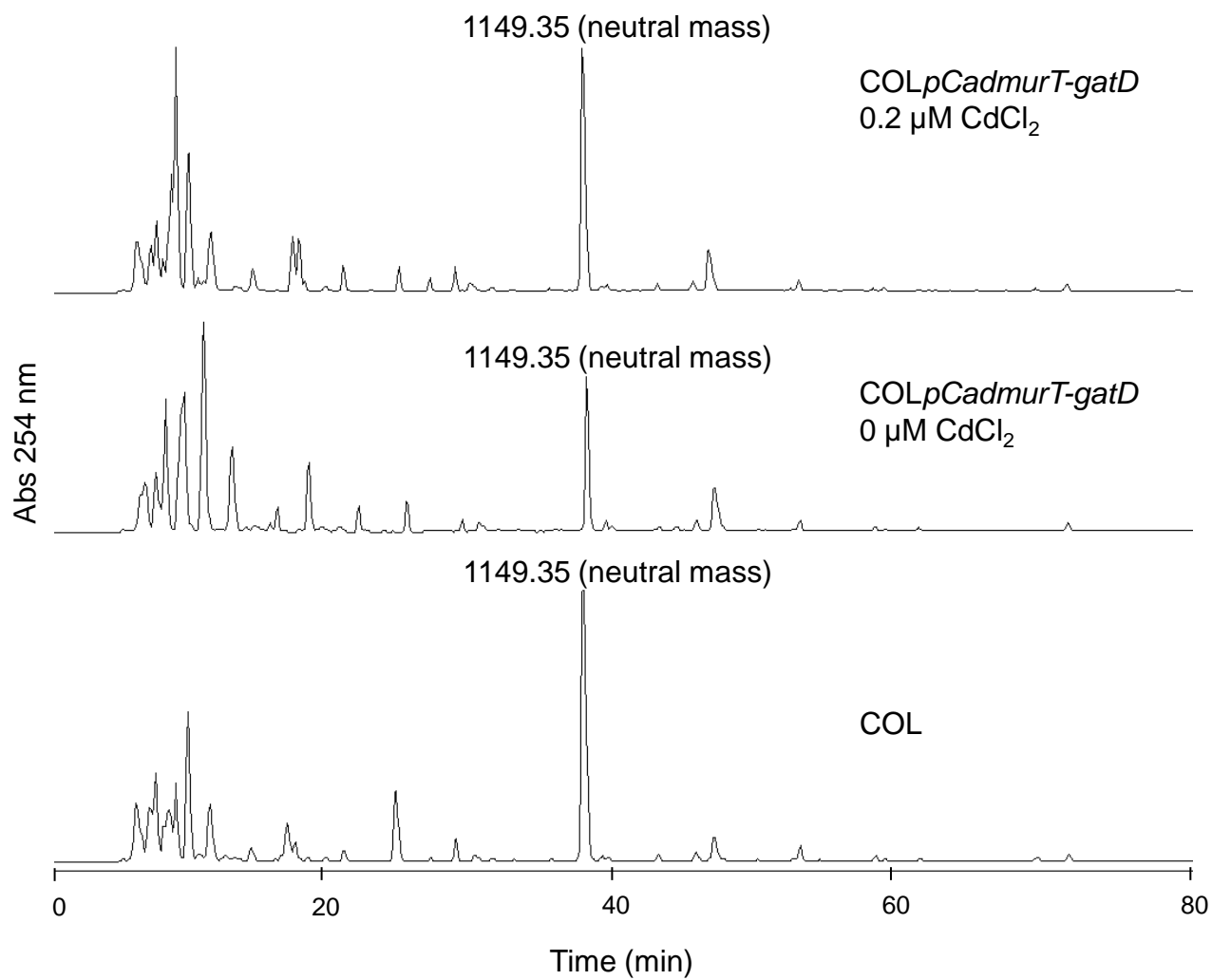

Supplement: Figure S4 — RP-HPLC profiles of UDP-linked precursor pools. The UDP-linked precursor pools of the train COL and COLpCadmurT-gatD grown with or without 0.2 µM of CdCl2. The major precursor structure (elution time of 38.0 min) was identified by mass spectrometry as UDP-MurNAc-L-Ala-D-iGlu-L-Lys-D-Ala-D-Ala. (PDF) [file ppat.1002508.s004.pdf]

Figure S5A

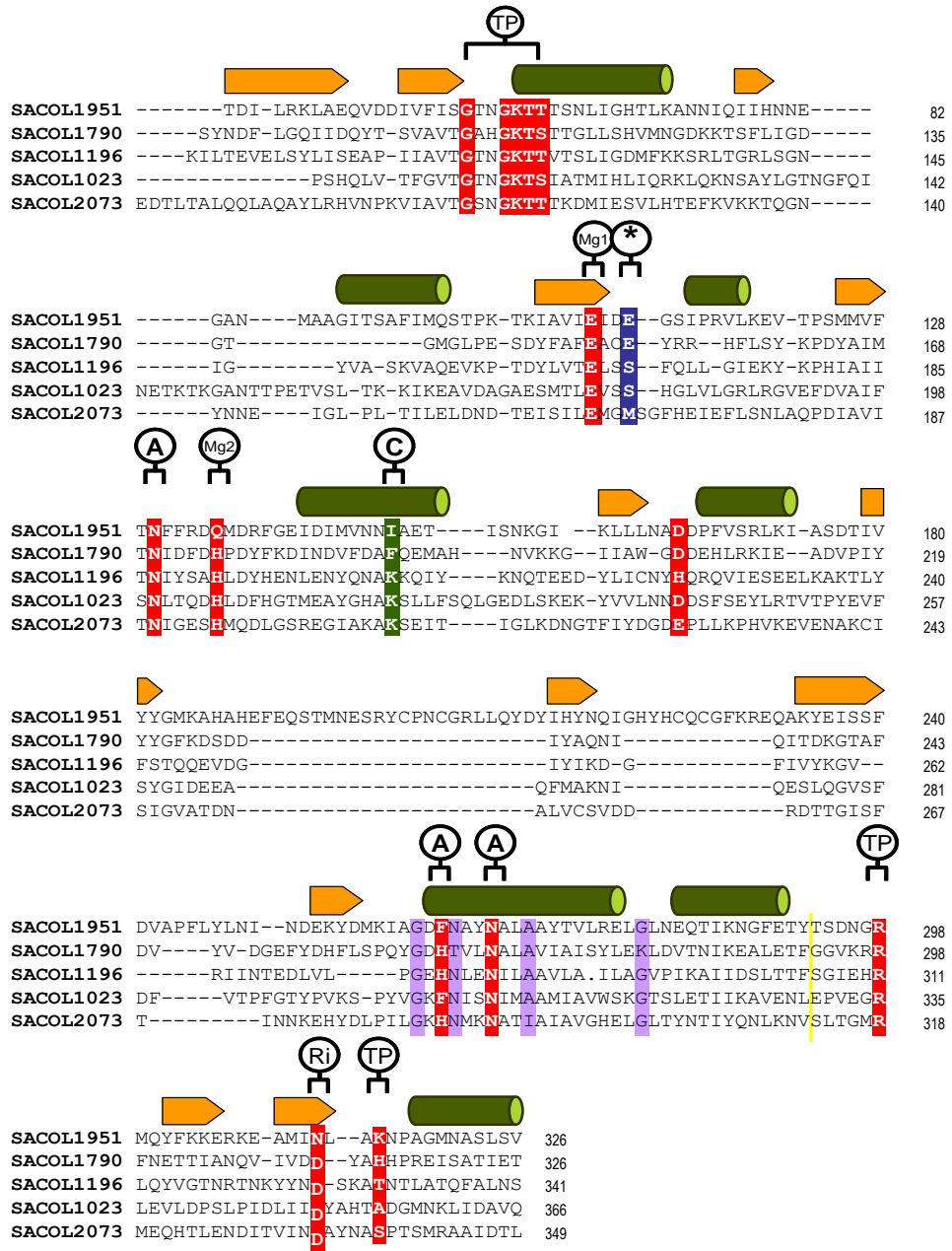

Figure S5B

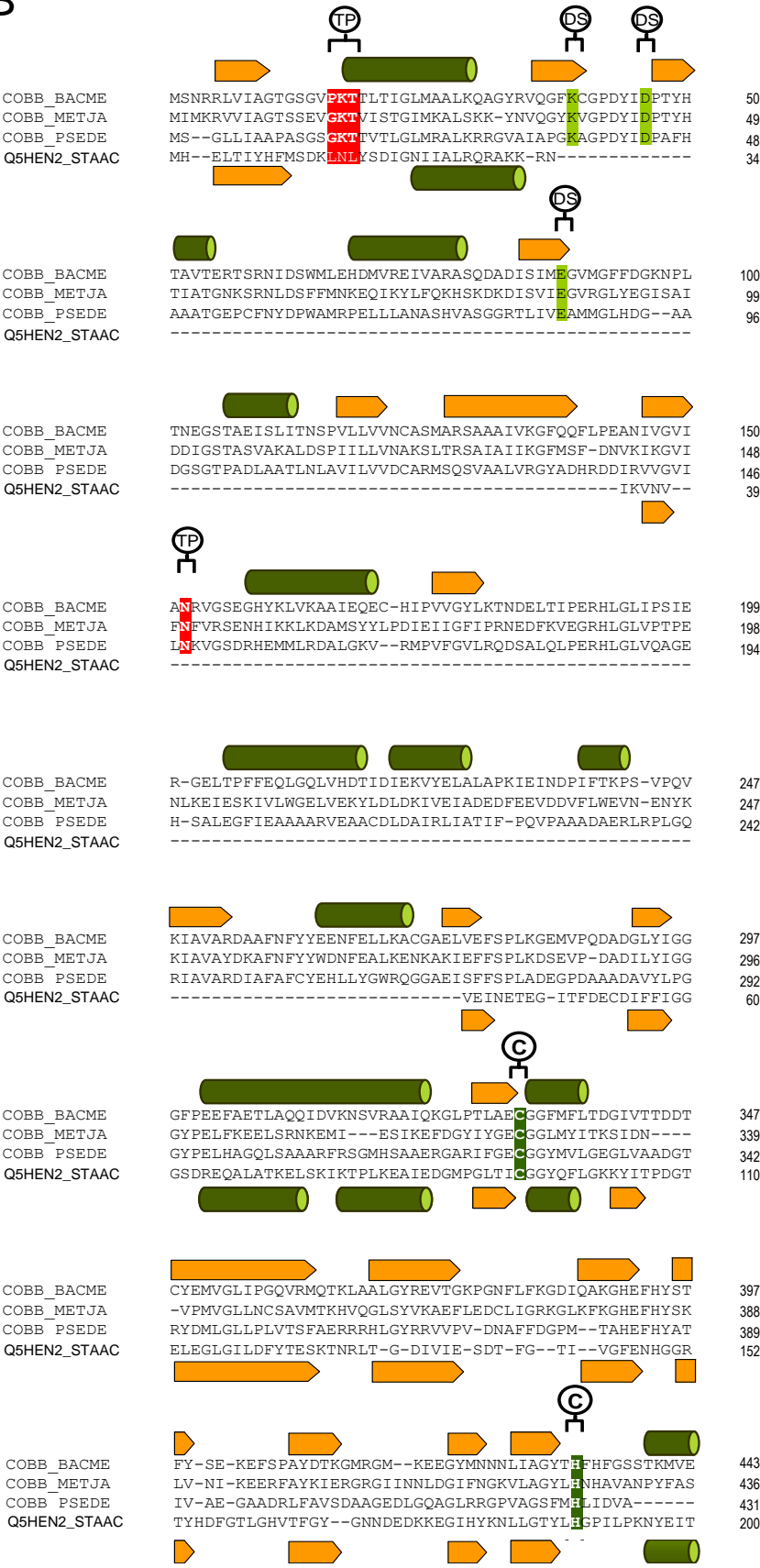

Supplement: Figure S5 — Structure-informed aminoacid sequence alignments. (A) Sequence alignmentof the central domain of Mur ligases. Residues involved with the nucleotide binding of four known S. aureus COL Mur ligases and MurT are labelled TP (ATP triphosphate), Mg1 and Mg2 (magnesium), A (adenine), and Ri (ATP ribose). The residue labelled C is the carbamoylated lysine residue observed in all the Mur enzymes except MurC; in this enzyme a glutamate residue, indicated with an asterisk (*) seems to play the same role in Mg2 coordination. The initial alignment was performed by TCoffee [7], the secondary structure was inferred for all sequences through Psipred [8], and the alignment was manually edited according to the latter.SACOL1951-MurT; SACOL1790-MurC; SACOL1196-MurD; SACOL1023-MurE; SACOL2073-MurF. In the top line α-helixes (green cylinders) and β-strands (orange arrows) were inferred for the sequences of the known Mur ligases. (B) Sequence alignment of the N-terminal halves of three known GATases. The residues involved in nucleotide binding of S. aureus COL GatD and three known GATases are indicated with a filled red box and are labelled TP (ATP triphosphate). Residues in green boxes marked with DS are deemed important for the dethiobiotin synthetase activity. The residues in the filled box labelled C are annotated as being directly involved with reactive center according to the GATase 3 (IPR011698) domain documentation. The initial alignment was performed by TCoffee [7], the secondary structure was inferred for all sequences through Psipred [8], and the alignment was manually edited according to the latter.GatD-Q5HEN2_STAAC (S. aureus); COBB_BACME (Bacillus megaterium); COBB_METJA (Methanocaldococcus jannaschii); COBB_PSEDE (Pseudomonas denitrificans). In the top line α-helixes (green cylinders) and β-strands (orange arrows) were inferred for the sequences of these three known GATases. In the bottom line, the same information is shown for GatD. (PDF) [file ppat.1002508.s005.pdf]
