# Supplementary material for: Identification of Genetic Determinants and Enzymes Involved with the Amidation of Glutamic Acid Residues in the Peptidoglycan of Staphylococcus aureus
Source: PLoS Pathog. 2012 Jan 26;8(1):e1002508. doi: 10.1371/journal.ppat.1002508 (PMC3267633; doi:10.1371/journal.ppat.1002508)
Supplement: Table S1 — Primers used in this study. (DOC) [file ppat.1002508.s006.doc]

|  | **Table S1. Primers used in this study** | | | |  | |
| --- | --- | --- | --- | --- | --- | --- |
| Primer | | | Sequence (5' - 3')^a^ | | | Source or  reference |
|  | | **Amplification of Transcripts (RT-PCR)** | | | |  |
|  | | P1949-R1 | | CTCTGAACATCGCATCAATGG | | This study |
|  | | P1948-R1 | | CGGGATCCCTAAACTACGGAGGGATGTG | | This study |
|  | | PmurT-D1 | | CTTCGGTGAAATTGATATTATGG | | This study |
|  | | PmurT-XR1 | | ATTGATCATCGCTTCTTTTCG | | This study |
|  | | PgatD-R1 | | GTGGAAGTGATAGAGAACAAGC | | This study |
|  | | PSK50f | | ACGCGTCGACGAACAATTAGAAGGCGA | | This study |
|  | | PmurT-D2 | | TATACATCAGACAATGGTCG | | This study |
|  | | PgatD-XR1 | | GCGCCTCGAGCGAGATTTCTTCTGTC | | This study |
|  | |  | |  | |  |
|  | | **Construction of the conditional mutant** | | | |  |
|  | | PmurT'F | | TCCCCCGGGCGAGTGGAAATTT**GAGG**AGG | | This study |
|  | | PmurT'R | | CGAGATCTGACCATTGTCTGATGTATACG | | This study |
|  | | PmurT-R1 | | GTTCTCTATCACTTCCACCACC | | This study |
|  | | PmurT-R2 | | GTGTTGATTGCATGATGAATGC | | This study |
|  | | PcadF | | GCACTTATTCAAGTGTATTT | | Novick |
|  | | PcadR | | GTTCAGACATTGACCTTCAC | | Novick |
|  | |  | |  | |  |
|  | | **Amplification of DNA probes (Northern blotting)** | | | |  |
|  | | PmurT-D | | TCCCCCGGGCGAGTGGAAATTTGAGGAGG | | This study |
|  | | PmurT-R | | CGAGATCTGACCATTGTCTGATGTATACG | | This study |
|  | | PgatD-D | | GTGGAAGTGATAGAGAACAAGC | | This study |
|  | | PgatD-R | | GAATACCCTTACGTTCACAAGC | | This study |
|  | | P1952-D | | GAATGTACGAGCGCCAAGTTC | | This study |
|  | | P1952-R | | CAATGGCAGCATACTGTGATAAAG | | This study |
|  | | FemC-D | | CTGGACAAGGTAAAGTTGCACG | | This study |
|  | | FemC-R | | CTAGTCCAGCTTCTAAGATTGC | | This study |
|  | |  | |  | |  |
|  | | **Construction of the complementation mutants** | | | |  |
|  | | PmurTSalI | | ACGCGTCGACATATGCGTGTGCTGC | | This study |
|  | | PmurTBamHI | | CGGGATCCATATTATGATTGACCTCCTTCAAAC | | This study |
|  | | PgatDSalI | | ACGCGTCGACGAACAATTAGAAGGCGA | | This study |
|  | | PgatDBamHI | | CGGGATCCAAATCCATTGATGCG | | This study |

^a^ The restriction sites included in the primers are underlined and the putative ribosome binding site is indicated in boldface type**.**
